# The Factory System and Factory Statistics

**Published:** 1837-01-01

**Authors:** 


### The Factory System and Factory Statistics.

The Quarterly Review, for the present month,* contains an article on the factory system, founded on as many as nine distinct reports or works published on that important subject. Of the nine documents, three are official, and the result of the labours of Commissioners of the King, or of Committees of the House of Commons. Two are productions of medical men, Dr. Kay and Mr. Charles Wing. Two emanate from Members of the House of Commons—the late Mr. Sadler and Mr. Fielden. And two are anonymous; one of these, termed "A Voice from the Factories," being in verse. All prove the interest taken by the public, the profession, and the legislature, in a system which involves the well-being of body and of mind, the happiness and lives, to say nothing of the eternal welfare, of many millions of our fellow-creatures.

There are two points of view in which we must look at the factory system—a political one and a medical. The political question is, no doubt, beset with many difficulties, and the economists are far from agreed upon it. Humanity revolts at the forced and unnatural labour of children: but they exist in numbers which, without such means of employment, could not possibly procure the necessaries of life. This very system has called them into being, and without it England could not sustain her enormous manufacturing population.

As medical men, we may discard the considerations of political economy, and examine the factory system as one affecting the public health. Our opinions have been asked by the Legislature and the community, and both have referred to us as the most appropriate judges, on one point at least of the issue. We have been appealed to, as philosophers and as philanthropists, by the friends of the factory child, and our feelings have been enlisted in the cause of the seemingly oppressed. If some of us have allowed our judgment to be a little warped by compassion, and have become partizans rather than umpires, the ancient and honourable character of the profession for humanity has been maintained, and the sin committed on *its* side must be deemed a venial one.

In the present short notice of the influence of the factories on health, we do not intend to discuss the whole question, but simply to exhibit a few of the facts. At a future opportunity we may consider it more fully. In this instance, we shall content ourselves with making use of the data, supplied by the industry and zeal of our contemporary.

A slight sketch of the history of factory labour may not be unacceptable. While it shews the unrelenting inhumanity of avarice, it displays the difficulties with which philanthropy and benevolence must struggle, before they can effect their noble ends. The system of employing children as factory labourers was the result of Arkwright's inventions. His looms took manufacturers out of the cottages and farm-houses, and assembled them in the counties of **Derbyshire**, Nottinghamshire, and more particularly in Lanca-

---

* December, 1836.

shire, where the machinery was used in large factories, built on the sides of streams capable of turning the water-wheel. There was an immediate demand for hands, and children were found to be best adapted for the work. The custom instantly sprang up of procuring apprentices from the different parish workhouses of London, Birmingham, and elsewhere. Many thousands of children, between the ages of seven and of fourteen years, were carted to the North. The horrors of this apprentice-system appear to have been dreadful. But it is past, and we need not rake them up again. But one fact may be stated. An agreement was made between a London parish and a Lancashire manufacturer, stipulating that, with every twenty sound children, one idiot should be taken !

The toil, the scourging, the crowding, the filth of the receptacles for the apprentices, reached such a height, that these outrages on nature were taken in hand by Nature herself, and pestilential fevers became alarmingly destructive. The public was roused, and a board of health was instituted at Manchester, and made its report in 1796. That report was highly damnatory of the factory system.

In 1802, the late Sir Robert Peel introduced and carried his first bill for the Relief of the Apprentices. It did relieve them, but steam being now applied to manufactures, the site of factories was removed from the sides of streams to towns and populous districts, where labour could be readily obtained, and where apprentices were an useless incumbrance. The bill afforded no protection for the children now employed, and, in 1815, Sir Robert proposed a second measure, which was not carried till the Session of 1819. Sir Robert had limited the hours of actual labour to eleven, for all persons between the ages of nine and sixteen. The Commons would not hear of eleven hours as an ultimatum, and extended the number to twelve.

It is worth while to pause for an instant, and listen to some of the medical evidence adduced by the master-manufacturers, in defence of an extended period of labour. To say the least of it, it is neither calculated to increase the public estimation of the logic, the candour, or the humanity of the profession. Fortunately, they who gave such evidence as we shall quote were few, and, both in numbers and in character, the medical advocates of moderate labour exceeded them.

"Edward Hulme, M.D. of Manchester.—Q. Suppose I were to ask you, whether you thought it *injurious to a child to be kept standing three-and-twenty hours out of the four-and-twenty*, should you not think it must necessarily be injurious to the health, without any fact to rest upon, as a simple proposition put to a gentleman in the medical profession? *A.* Before I answered that question I should wish to have an examination, to see how the case stood.—Q. Would it be injurious to a child, in your judgment, as a medical man, if, at the time he got his meals, he was still kept engaged in the employment he was about? *A.* *Those* are questions *which I find a great difficulty in answering*" ! !

Q. I ask you, as a medical man, whether, supposing a person, during the time he was eating his meals, was employed in manual labour, is it your judgment that the food will be as nutritious to him as it would be if he were unemployed? *A.* I should imagine that the food would be equally nutritious to him, *if he did the manual labour of handling his knife and fork*" ! ! !

It does appear marvellous how any physician, surgeon, or apothecary could make such answers to such questions. Dr. Hulme could not determine, as an abstract proposition, whether standing twenty-three hours out of the twenty-four, would or would not be prejudicial to the health of a child! He wished to examine how the case stood! He must have been indeed a lover of the Baconian method. The replies are absolutely farcical.

"Mr. Ainsworth, surgeon.—Can a child from six years of age to twelve be employed from thirteen to fifteen hours daily in a temperature of 80°, and in an erect position, consistently with safety to its constitution? I never saw an instance of the kind as a fact brought before me, and therefore cannot say.—Am I to understand you, *as a medical man*, can *give no opinion*, whether it would not be more exhausting to the human body to keep in *an erect position* for twelve hours, than in a *reclining* position? *I have no facts to lead me to conclude.*

Thomas Turner, surgeon.—Do you think it would benefit a child's health of eight years old to be kept twelve hours upon his legs? *Really I am not prepared to answer that question.*—What do you think of it? I really cannot tell you. —You can form no opinion, whether to a child of eight years of age being kept standing fourteen hours without intermission would be injurious to his health or not? I have no facts to guide me." 404.

It would be idle to expatiate on the character of such testimony as this. We hope we may have no more of it. Medical men are notoriously bad witnesses, but evidence of the nature we have quoted is more than usually absurd. Whilst some gentlemen experienced such extreme difficulty in determining whether factory labour was injurious—whilst Dr. Hulme thought work during meals wholesome provided the knife and fork were worked too, —while Mr. Ainsworth was unable to decide whether standing for twelve hours was more exhausting than lying down—and while Mr. Turner really could not tell if he thought or did not think that the health of a child of eight years old would be benefited by his being twelve hours on his legs— the actual nature of the factory-labour was as follows:—

It was in nearly all the cotton-mills of Lancashire and its neighbourhood, excepting Saturday, from thirteen to sixteen hours a day, inclusive of one hour, or less, *nominally* allowed for dinner. Many of those subjected to such labour were children of nine, eight, seven, and six years of age, and previously to the stirring of the investigation, under six, and, in some instances, *under five!* The children continued constantly at work so long as the machinery was in motion, during which time they were not permitted to sit down or to leave the factory. They often complained (naturally enough, our readers will think) of fatigue, and aching limbs; in this state of exhaustion, towards the close of the day, they were beaten by the spinners, or overlookers, or even by their own parents, that blows might supply the deficiency of strength. In most cotton-factories, during the greater part, and often the whole of the time *nominally* allotted for dinner, the children were occupied in cleaning the machinery; no time was allowed for the breakfast or afternoon meals, which were snatched in mouthfuls during the progress of uninterrupted labour; the refreshment not unfrequently remaining untouched till it became cold, and covered with dust and dirt from the cotton-flyings. It appeared, moreover, that the temperature in many cotton-mills was from 75° to 80°, in others from 80° to 85°, and occasionally as high as 90°.

Such was the state of things when, in 1819, the twelve hours' Bill was carried. After the lapse of a few years, Sir John Hobhouse introduced an eleven hours' Bill, which was burked. He obtained, however, an Act in 1825, which limited the labour of persons under sixteen to sixty-nine hours in the week, twelve on five days and nine on Saturdays. But his bill comprised the cotton-factories only. In 1831 it was somewhat improved by the prohibition of night-work for all under twenty-one, and by the advance of the ages entitled to protection from sixteen to eighteen years. It is obvious that while something had been gained, humanity had yet much to effect, before she could be satisfied. Mr. Sadler, a very eloquent and a very earnest advocate of the cause of the factory-child, first appealed to the country in 1831, and brought in his Bill in the ensuing Session. The measure was cushioned, and Mr. Sadler never was in Parliament again. A Committee, which was appointed on the introduction of the Bill, elicited a mass of evidence, which attracted the attention, and greatly excited the feelings of the public. A Commission was now appointed by the Crown, and the Commissioners, four of them medical, were directed to investigate personally and systematically the condition of the children. Their Report was presented to the House of Commons in July, 1833. In March of that year, Lord Ashley had moved the first reading of a Bill, resembling in all essential particulars, that of Mr. Sadler. It sought to restrict the labour to a period of ten hours, for all ages from nine to eighteen. The second reading of the Bill was postponed until after the presentment of the Report, which, as we have observed, was in July. The Report, in question, recommended eight hours as the ultimatum of labour for young children, and on that recommendation was based the law which is now in operation. The law in question, Lord Ashley's proposition being superseded, enacts:—

A period of eight hours for all under thirteen, and one of twelve for those between thirteen and eighteen. By a clause, it was provided that the Act should come into operation by parts, each in succession. At the end of six months, children under eleven were to enjoy the benefit of the eight hours' limitation; at the end of eighteen, all under twelve; and at the end of thirty, all under thirteen. Subsequently, the Ministry endeavoured to vitiate the Act, but met with a defeat, and it is at present the law of the land. There is every reason, however, for believing that a ten hours' Bill for all ages will at no great distance of time be introduced, and as medical evidence has throughout been properly depended on as the basis of any legislative enactments, we have laid before our readers this sketch of the history of the various measures that have been attempted or adopted.

It only remains for us to notice some of the facts adduced, to shew the actual working of the factory system.

Dr. Ray, in a pamphlet, intituled, "The Moral and Physical Condition of the Working Classes employed in the Cotton Manufacture in Manchester," after presenting an elaborate survey of the streets, and the houses inhabited by the operatives—and after painting in the darkest colours the imperfect ventilation and the filth of all, introduces us to the person and the family of a cotton-spinner. Let us accompany the Doctor down a lane containing "heaps of refuse, deep ruts, stagnant pools, ordure, &c." and opening the door of what is facetiously called a house, let us gaze upon the inmates:—

"The population employed in the cotton-factories," continues the Doctor, "rises at five o'clock in the morning; works in the mills from six till eight o'clock, and returns home for half an hour or forty minutes, to breakfast. This meal generally consists of tea or coffee, with a little bread. Oatmeal porridge is sometimes, but of late, rarely used, and chiefly by the men; but the stimulus of tea is preferred, and especially by the women. The tea is almost always of a bad, and sometimes of a deleterious quality; the infusion is weak, and little or no milk is added. The operatives return to the mills and workshops until twelve o'clock, when an hour is allowed for dinner.

Amongst those who obtain the lower rates of wages, this meal generally consists of boiled potatoes. The mess of potatoes is put into one large dish; melted lard and butter are poured upon them, and a few pieces of fried fat bacon, are sometimes mingled with them, and but seldom a little meat. Those who obtain better wages, or families whose aggregate income is larger, add a greater proportion of animal food to this meal, at least three times in the week; but the quantity consumed by the labouring population is not great. The family sits round the table, and each rapidly appropriates his portion on a plate, or, they all plunge their spoons into the dish, and with an animal eagerness satisfy the cravings of their appetite. At the expiration of the hour, they are all again employed in the workshops or mills, where they generally again indulge in the use of tea, often mingled with spirits, accompanied by a little bread. Oatmeal or potatoes are, however, taken by some, a second time in the evening." 420.

During twelve hours of the day, the operative drudges, in a heated atmosphere, watching the movements, and assisting the operations of a steam engine. His toil is incessant, and rational relaxation he can have none, because, when the hours of work are over, bodily fatigue precludes every thing save indulgence in spirits, in the grossest sensuality, or in the indolence of mere exhaustion. What wonder then, that the factory labourers have become as a class degenerate in body—debased in mind—a grovelling, gin-drinking, demoralized set, whose animal vices are painted in the most revolting hues by those who have narrowly watched their habits. We have not room for the statements of Dr. Kay and of Mr. Gregg on this head, nor do they so much concern us, as the merely physical effects of labour.

It appears that the whole number of persons employed in cotton-factories in the year 1835, was 220,134, four-times as many as in 1818. Of that number, 94,287 were between the ages of eight and eighteen years—and 125,877, above the latter age.

It is singular to observe the effects of the improvements in machinery on the labour of the operatives. It will be remembered that in 1819, the Legislature declared that twelve hours of mill-work were sufficient. The following calculation serves to exhibit the amount of labour undergone when that enactment was carried.

'A table shewing the distance over which a piecer had to travel, in following a pair of mules spinning cotton-yarn of Nos. 40, in the year 1815.

| The spinner put up 820 stretches daily, on each of 2 mules, 12 yards long each, and was attended by 3 persons. | Number of stretches daily. | Number of yards from mule to mule. | Number of yards comprising the piecer's work along each mule, and over which he must walk each stretch. | Total Number of yards. | Distance in miles. |
|---|---|---|---|---|---|
| | 1640 | 5 | 4 | 14,760 | 8 and a fraction.' |

The operation of the bill would appear to have done any thing but diminish the spinner's toil. The ingenuity of cupidity travelled faster than the legislation of humanity, as the next statement will demonstrate.

'A table shewing the distance over which a child must walk, in following a pair of mules spinning cotton-yarn of No. 40, at Manchester, in 1832.

| The spinner puts up daily 2200 stretches on each of 2 mules. | Number of stretches daily. | Number of yards from mule to mule. | Average number of yards which a child walks along each mule per stretch. | Total Number of yards daily. | Distance in miles. |
|---|---|---|---|---|---|
| | 4400 | 5 | 3 | 35,200 | 20.' |

At Bolton-le-Moors, the wheels would appear to revolve with still more astonishing rapidity, and it is estimated that a piecer, in attending a pair of mules spinning cotton-yarn, must there travel daily twenty-five miles! All this is done in a heated and impure atmosphere, by persons indifferently fed—an irksome daily drudgery, performed under the stimulus of absolute necessity. That the lower classes in a manufacturing country, or indeed in any, must either work or starve is true, and an exaggerated and preposterous compassion may be entertained on their behalf. But where the numbers of the labourers are such that the competition among them resulting from necessity, disables them from defending themselves against the exactions and tyranny of capital, both humanity and policy demand the interference of the nation, and the effectual protection of Parliament.

If such reasoning is applicable to the case of adults, how much more forcibly must it apply to children—to those helpless creatures consigned by inhuman parents to almost as inhuman overlookers—who are made to taste the most sickening toil and care at the only age which nature has intended to be free from them. We cannot effect the Utopian project of releasing the poor man's child from labour, but we can and we ought to make that consistent with the powers of childhood. As medical philosophers we are called on to determine what those powers are, and what amount of work is consistent with the integrity of the infantile constitution. As medical practitioners we have been summoned to report, and probably we shall be summoned again, on the actual results of the existing system. The evidence that we have given has not always been calculated to exhibit either our candour, our science, or our good feelings, and we may be sure that the intelligent portion of the public both remarks and remembers these disadvantageous exhibitions.

It seems that the tendency of the factory system is to substitute more and more exclusively, the labour of the young for that of the adult. There are many apparently good reasons for this, on which, however, we need not insist, as the fact is all we want. The consequence is, that the old are thrown on the young for support, the reverse of the order of nature. A sad picture has been drawn by too many observers of the subsistence of parents on offspring. That, however, is a consideration which belongs to the moral, rather than the medical philosopher, and we dismiss it. It concerns us more to hear that—

"The number of operatives above the age of forty is incredibly small. We must refer our readers to a curious but authentic document, arranged about the year 1831, during a great 'turn out,' from forty-two mills in Mosley, Ashton, Staley-Bridge, and Dukinfield, of 1665 persons, whose ages ranged from fifteen to sixty. Of these 1584 were below forty-five; *three* only had attained a period between fifty-five and sixty; and not more than fifty-one between forty-five and fifty were counted, as fit for work! Mr. M'Nish, a witness entitled to the utmost credit, even by the admission of the Commissioners, and in truth, as every one must perceive upon reading his evidence, a man of singular sagacity, deposed, in the year 1832, that, by actual enumeration of 1600 men in the factories of Renfrew and Lanark, he ascertained that not more than ten had reached forty-five years of age, and these, he added, were retained by the special indulgence and humanity of their masters. The spinners at that period are so broken down that they cannot produce the required quantity. 'Their eye-sight fails,' and then they are turned off, and younger men employed."

We now quit this subject. We have drawn the attention of our readers to it, because it is evidently still unsettled, and will again occupy the attention of the Legislature. Medical evidence may once more be requisite, and it is well for the profession to be put in possession of the general facts, which will enable it to form a more comprehensive and more accurate estimate of the various bearings of the question at issue. Independently of this, which may be said to be in some measure a personal consideration, it is daily becoming more indispensable for the medical practitioner to be well informed, on matters which affect the health of large sections of the nation. His opinions are frequently publicly asked, and if inconsiderately or ignorantly expressed, they redound to his own disgrace, and to the shame of the profession.

